# A 4NsL chromosome segment from *Leymus mollis* confers stripe rust and Fusarium head blight resistance in wheat

**DOI:** 10.3389/fpls.2025.1711424

**Published:** 2025-12-01

**Authors:** Xin Du, Yanlong Jin, Wei Ren, Dongdong Ge, Xiaomin Xu, Xiaoqi Zhao, Yanzhen Wang, Tingting Li, Jixin Zhao, Changyou Wang, Tingdong Li, Chunhuan Chen, Xinlun Liu, Pingchuan Deng, Wanquan Ji

**Affiliations:** 1 State Key Laboratory of Crop Stress Biology in Arid Areas, College of Agronomy, Northwest A&F University, Yangling, Shaanxi, China; 2 Institute of Crop Research, Anhui Academy of Agricultural Science, Hefei, China; 3 College of Agriculture, Shihezi University, Shihezi, Xinjiang, China; 4 Center for Agricultural Genetic Resources Research, Shanxi Agricultural University, Taiyuan, Shanxi, China; 5 College of Bioengineering, Yangling Vocational Technical College, Yangling, Shaanxi, China

**Keywords:** *Leymus mollis*, translocation line, specific markers, stripe rust, fusarium head blight

## Abstract

Leymus mollis (Trin.) Pilg. (2n = 4x = 28, NsNsXmXm) serves as a tertiary genetic pool for wheat improvement, harboring valuable traits such as disease resistance and stress tolerance. To harness this potential, we characterized two novel wheat-L. mollis translocation lines, M892 and M956, derived from a cross between common wheat and a 4Ns disomic addition line. Phenotypic evaluation revealed that M956 displays high resistance to both stripe rust and Fusarium head blight, while M892 is susceptible; both lines exhibit enhanced grain weight, with M892 additionally showing a dwarf phenotype. Cytogenetic analyses identified M892 as a T5DL-4NsL-5DL·5DS insertional translocation line and M956 as a T4NsL-5DL·5DS terminal translocation line. Genetic mapping localized the stripe rust resistance to a critical 98 Mb region on chromosome 4NsL, and comparative transcriptomics pinpointed 47 candidate resistance genes uniquely upregulated in M956. Furthermore, we developed eight 4NsL-specific molecular markers, providing a practical tool for the marker-assisted utilization of these resistance genes in wheat breeding programs.

## 1 Introduction

Wheat (*Triticum aestivum* L.), is a cornerstone of global food security, and makes a substantial contribution to worldwide caloric intake (Braun et al., 2010). However, its productivity faces persistent threats from devastating pathogens, particularly stripe rust (*Puccinia striiformis* f. sp. *tritici*), powdery mildew (*Blumeria graminis* f. sp. *tritici*), and Fusarium head blight (FHB, *Fusarium graminearum*). These diseases collectively cause annual yield losses of 20-80%, quality deterioration, and dangerous mycotoxin contamination, posing critical challenges to sustainable production of wheat (Chen et al., 2005; Figueroa et al., 2018).

Traditional breeding strategies that rely on the introgression of resistance genes from closely related species face limitations due to the lack of genetic diversity in the wheat gene pool. This limitation has driven the exploration of novel resistance traits in distantly wild relatives. *Leymus mollis* (Trin.) Pilger (NsNsXmXm, 2*n* = 4*x* = 28), a perennial tetraploid *Triticeae* species endemic to coastal and arid ecosystems, is an exceptional germplasm resource. It exhibits broad-spectrum resistance to major wheat diseases (stripe rust, FHB, powdery mildew, and leaf rust) as well as desirable abiotic stress tolerance (Fatih, 1983; Kishii et al., 2003; Fan et al., 2009; Habora et al., 2012), making it particularly valuable as tertiary genetic material for wheat improvement.

Distant hybridization between wheat and *L. mollis* is a promising strategy for improving disease resistance. Seminal work in China’s 1980s achieved the first wheat-*L. mollis* F_1_ hybrids by embryo rescue and colchicine-mediated chromosome doubling using common wheat cv. 7182 and *L. mollis* (Chen et al., 1985). The subsequent produced stable alien addition and substitution lines with heritable resistance to stripe rust and powdery mildew via chromosome engineering (Yang et al., 2014; Zhang et al., 2017; Du et al., 2022; Feng et al., 2022). Meanwhile, *L. mollis* contains genes resistant to FHB and is one of the few sources of resistance to FHB in wheat (Chen et al., 2005; Qi et al., 2008; Zhao et al., 2019; Du et al., 2022).

Accurate detection of introgressed chromatin remains crucial for effective utilization of alien gene. Cytological techniques, particularly genomic *in situ* hybridization (GISH) and fluorescence *in situ* hybridization (FISH), are indispensable tools for visualizing whole and segmented chromosomes (Du et al., 2022; Feng et al., 2022). While these approaches provide essential cytogenetic verification, molecular markers offer high resolution in detecting introgressions. Early marker systems including restriction fragment length polymorphisms (RFLPs), random amplified polymorphic DNA (RAPD), simple sequence repeats (SSRs), expressed sequence tags (ESTs), and PCR-based landmark unique gene (PLUG) markers played pivotal roles in initial identification of wheat hybrids (Sharp et al., 1989; Peil et al., 1997, 1998; Yang et al., 2015). However, their limited specificity and throughput restricted comprehensive alien chromatin analysis.

The latest advancements in high-throughput sequencing have revolutionized the development of molecular markers. Specific-locus amplified fragment sequencing (SLAF-seq) (Chen et al., 2013; Song et al., 2020; Wang et al., 2020, 2022), genotyping-by-sequencing (GBS) and transcriptome sequencing have greatly improved detection accuracy and efficiency (Li et al., 2017; Wang et al., 2018; Li et al., 2019a, 2019c; Singh et al., 2020). Nevertheless, the advancement in developing verified, polymorphic, and user-friendly molecular markers specific to *L. mollis* chromosomes introgressed into wheat remains limited. Currently, only a small number have been reported for specific chromosome arms (e.g., 13 SLAF-seq markers for *Lm*#2Ns and 16 RNA-seq markers for *Lm*#4Ns).

The emergence of high-density wheat SNP arrays (9K, 15K, 35K, 55K, 90K, 660K and 820K) have revolutionized wheat genetic research (Sun et al., 2020). The 15K and 55K arrays, exploiting homologies between wheat and its wild relatives, have successfully detected chromatin transfers from *Psathyrostachys huashanica*, (Li et al., 2019b) *Leymus mollis* (Li et al., 2021; Du et al., 2022) and *Thinopyrum* species (Wang et al., 2020, 2022). However, these platforms exhibit limitations in resolving small alien segments and mining introgressed genes. Third-generation genotyping platforms, such as Genotyping by Target Sequencing (GBTS), which integrats target capture with high-throughput sequencing, marks a paradigm shift (Guo et al., 2019). The wheat-*Psathyrostachys huashanica* liquid-phase array (GenoBaits^®^WheatplusPh) accurately characterized the wheat-*L. mollis* 4Ns(4D) disomic substitution line (Du et al., 2022). In addition, the GenoBaits^®^WheatplusEE array enabled breakpoint analysis in *Thinopyrum* introgressions (Deng et al., 2024).

Current strategies for translocation generation include CS *ph1b* manipulation, ^60^Co γ irradiation, gametocidal chromosome systems from *Aegilops*. Despite these advances, few wheat-*L. mollis* translocation lines have been reported, highlighting the urgent need for accelerated germplasm development. Therefore, to facilitate the utilization of the disease resistance genes located on the *L. mollis* 4Ns chromosome, this study aimed to: (1) create and identify novel wheat-*L. mollis* translocation lines via the gametocidal chromosome; (2) precisely characterize their chromosomal structures using integrated molecular cytogenetic and genomic approaches; (3) evaluate their resistance to stripe rust and FHB, as well as key agronomic traits; and (4) delineate the resistance-associated chromosomal segment and develop specific molecular markers for breeding applications.

## 2 Materials and methods

### 2.1 Plant materials

All materials (**Supplementary Table 1**) were maintained at the College of Agronomy, Northwest A&F University, Yangling, China. The common wheat cultivars ‘7182’ (AABBDD, 2*n* = 42) and ‘Nonglin 26’ (NL26) served as recurrent parents. The wild relatives *L. mollis* and *Psathyrostachys huashanica* Keng ex P.C.Kuo (2*n* = 14, NsNs) were used as alien gene donors and for probe preparation, respectively. The key experimental materials were wheat-*L. mollis* derivatives: the Lm#4Ns(4D) disomic substitution line M862, and the two novel translocation lines (M892 and M956) under investigation, which were selected from the progeny of a cross between the 4Ns disomic addition line M852 and an NL26 line carrying the gametocidal chromosome 3C from *Aegilops triuncialis*. Control cultivars ‘Huixianhong’ (HXH), ‘Xiaoyan 22’ (XY22), and ‘Sumai 3’ (SM3) were used for disease resistance assays.

### 2.2 GISH and sequential FISH-GISH analyses

GISH was performed to detect the *L. mollis* chromatin using a modified protocol (Fu et al., 2012b). Root tip chromosome preparations were hybridized with fluorescein-12-dUTP-labeled *L. mollis* genomic DNA and Texas Red-5-dCTP-labeled *P. huashanica* genomic DNA. The oligonucleotide probes Oligo-pSc119.2 (6-FAM-5′, green) and Oligo-pTa535 (TAMRA-5′, red) were used for sequential FISH-GISH analysis on the same slide (Li et al., 2020). All probes were synthesized by Invitrogen Biotechnology Co., Ltd (Shanghai). Chromosomes were counterstained with 4,6-diamidino-2-phenylindole (DAPI), and visualized using an Olympus BX63 epifluorescence microscope equipped with a DP80 CCD camera (Olympus Corporation, Tokyo, Japan). Image analysis was performed using CellSens Dimension 1.16 software.

### 2.3 Wheat-*P. huashanica* liquid array analysis

The homoeologous group chromosomes of M892 and M956 was characterized using wheat-*P. huashanica* 45K liquid array (GenoBaits^®^WheatplusPh), which integrates 90,000 capture probes (10,000 from wheat and 80,000 from *P. huashanica*). The probe was synthesized at MolBreeding Biotechnology (Shijiazhuang, China). Raw reads (HiSeq X) were evaluated using FastQC v0.10.1 (De Sena Brandine and Smith, 2019). Trimmomatic was used to filter out adapters and low-quality reads (Bolger et al., 2014). Clean reads were aligned to the wheat reference genome (IWGSC RefSeq v2.1) using the BWA (Li, 2012). Probe sequence depth was quantified using bamdst v1.0.6 and visualized through R scripts.

### 2.4 Evaluation of agronomic traits

Comprehensive agronomic evaluations were conducted for M892, M956 and their parental cultivars (7182, NL26) during two consecutive growing seasons (2020-2021 and 2021-2022). At physiological maturity, ten representative plants per genotype were randomly selected for comprehensive trait evaluation (plant height, tiller number, spike length, spikelets number, florets number per spikelet, kernel number per spike, thousand-kernel weight, kernel length, kernel width and awnedness). Grain size was measured using the SC-G automated seed testing system (Wseen Detection Technology Co., Ltd., Hangzhou, China), with measurements of length and width taken on 200-250 individual grains per genetic line across five biological replicates. Statistical processing was performed in IBM SPSS Statistics 23 (IBM Corp., Armonk, NY, USA). The least significant difference (LSD) test at α = 0.05 probability level was applied for *post-hoc* comparisons between genotypes after confirming significant differences (p < 0.05) through one-way ANOVA.

### 2.5 Identification of resistance to stripe rust and FHB

*Puccinia striiformis* f. sp. *tritici* (*Pst*) races CYR32 and CYR34 were utilized to identify stripe rust resistance at the seedling-stage, and mixed races (V_CYR32_/V_CYR33_/V_CYR34_ = 1:1:1) was employed at the adult-stage. FHB resistance was evaluated using *F. graminearum* strain PH1. All inoculums were provided by the College of Plant Protection, Northwest A&F University, China.

The reactions of M892 and M956 to stripe rust were systematically evaluated at both adult and seedling stages. Field evaluations of adult-plant resistance were conducted during two consecutive growing seasons (2020-2021 and 2021-2022) using a mixed *Pst* races (CYR32, CYR33, CYR34). Inoculations were performed at the jointing stage using a standardized shaker-applied spore powder method (Wang et al., 2020). Experimental design including three biological replicates was performed, and using susceptible controls (HXH, XY22) and parental lines (7182, NL26) as reference genotypes.

Seedling-stage evaluation were conducted under plant incubator (25 ± 1°C, 16 h/8 h photoperiod, 70 ± 5% RH) using individual race inoculations (CYR32, CYR34). Two-leaf stage seedlings were spray-inoculated with fresh urediniospore suspensions (1×10^5^ spores mL^-1^ in 0.01% Tween-20 aqueous solution) following established protocols (Du et al., 2022). Infection types (IT) were evaluated using a standardized 0-4 scal (Kang et al., 2002), where IT 0-2 (chlorotic/necrotic flecks without sporulation) indicated resistance and IT 3-4 (abundant uredinia with susceptible necrosis) denoted susceptibility. Observations were recorded at 4-day intervals until 21 days post-inoculation (DPI), coinciding with complete susceptibility expression in HXH controls, with secondary scoring conducted one week after initial evaluation to confirm disease progression patterns.

Type II resistance (post-infection spread limitation) to FHB was quantified via single-floret inoculations with strain PH1 across two crop seasons (2020-2021 and 2021-2022) (Guo et al., 2015). Inoculations were performed at anthesis by injecting 10 μL aliquots of 2.5×10^5^ spores·mL⁻¹ suspension into the fifth floret of central spikelets, with ten replicate spikelets per genotype. Control experiment consisted of the susceptible cultivar XY22 and resistant line SM3. Inoculated spikelets were hydrated with mist irrigation and enclosed in transparent polyethylene bags (72 h, >95% RH). Disease progression was evaluated at 21 DPI using three complementary metrics: the infected spikelet rate (ISR, %), severity level (SL) on a 0-4 scale, and the resistance evaluation (RE) categories (I: immune, R: resistant, MR: moderately resistant, MS: moderately susceptible, S: susceptible). All assessments adhered to the Agricultural Industry Standard of China (NY/T 1443.4-2007) for wheat FHB resistance.

### 2.6 Library construction and RNA sequencing

M892, M956 and their parent cv. 7182 were inoculated with *Pst* race CYR32 at the two-leaf stage. Plants were sprayed with sterile water served as a mock control. RNA samples for Illumina sequencing were harvested at 24 h and 48 h from rust-inoculated, 0 h, 24 h and 48 h from mock-inoculated. RNA-seq was performed on the fresh and tender leaves collected 24 and 48 hours after pathogen inoculation, as well as on the control group at 0, 24, and 48 hours, with three biological replicates per condition.

Total RNA was extracted using TRIZOL reagent (Thermo Fisher Scientific Waltham, MA, USA) and assessed for integrity (RIN >7) using a 2100 Bioanalyzer (Agilent Technologies, Santa Clara, CA). Forty-five strand-specific libraries were constructed and sequenced on an Illumina NovaSeq 6000 platform with 150 bp paired-end reads by Biomarker Technologies Corporation (Beijing, China). Raw reads were processed through FASTX-Toolkit to remove adapter (Schmieder and Edwards, 2011), poly-N regions (>5%), and low-quality bases (Phred score <Q30). Quality assessment of clean reads was performed using FastQC (Andrews, 2013).

### 2.7 *De novo* assembly of Lm#4NsL and functional annotation

The *Triticum aestivum* (IWGSC RefSeq v2.1) genome was integrated with the *Puccinia striiformis* (Pst134E_v1_pri) genome to construct a mixture genome. High-quality reads were aligned against the mixture genome using Hisat2 (Pertea et al., 2016), generating mapped and unmapped reads. Subsequent analyses were divided into reference-based and *de novo* approaches. For the reference-based analysis, reads mapped to the stripe rust genome were systematically excluded, retaining only those aligned to the wheat genome for downstream investigations.

In the *de novo* assembly pipeline, unmapped reads from samples M892 and M956 were assembled using Trinity (v2.8.4) (Grabherr et al., 2011). Redundant sequences were reduced by 90% identity clustering using CD-HIT (v4.6.2) (Fu et al., 2012a), and the longest transcript isoforms were retained as unigenes. Quantitative analysis of unmapped reads from all accessions against assembled unigenes was performed using bowtie2 (v2.2.5) integrated with RSEM (v1.2.8) (Li and Dewey, 2011; Langmead and Salzberg, 2012). To ensure the fidelity of non-host transcripts, unigenes showing FPKM = 0 in parent 7182 but FPKM ≥1 in both M892 and M956 were retained. Subsequently, this curated unigene dataset served as the foundation for differential expression analysis, functional annotation of alien chromosomal elements, and molecular marker development.

Function annotation of alien unigenes was carried out via BLAST (Altschul et al., 1990) based homology searches against seven biological databases: NCBI NR (non-redundant proteins), Swiss-Prot (curated proteins), GO (Gene Ontology), KEGG (metabolic pathways), eggNOG (Non-supervised Orthologous Groups), COG (protein clusters), and Pfam (protein domains). In addition, differential unigenes (DEGs) were obtained using DESeq2 (Log2|fold change| ≥ 1 and padjust ≤ 0.05).

### 2.8 Development of Lm#4NsL specific molecular markers based on RNA-seq

Total DEGs comparison was conducted between M892 and M956. The unique DEGs of M956 were screened out and then compared with the genomes of *T. urartu* (AA), *A.* sp*eltoides* (BB), *A. tauschii* (DD), *Hordeum vulgare* (HH), *Secale cereale* (RR), *Thinopyrum elongatum* (EE), *A. longissima* (SS), *T. aestivum* (AABBDD), the unpublished genome of *P. huashanica* (NsNs), and the unpublished sequence library of *Leymus mollis*. Candidate Unigene sequences conforming to Lm#4NsL were screened according to the following criteria: (I) The similarity was 0% in the genomes except those of *P. huashanica* and *L. mollis*. (II) The similarity with the genomes of *P. huashanica* and *L. mollis* exceeded 50%.

Bulk primer design for the Lm#4Ns candidate unigenes was performed using the primer3 with default parameters (https://www.primer3plus.com/). All designed primers were subsequently screened for specificity by in silico alignment against the genomes of wheat, barley, and rye by the WheatOmics 1.0 platform (http://wheatomics.sdau.edu.cn/). A total of 80 primer pairs meeting the specificity criteria were randomly selected for commercial synthesis (AuGCT Biotechnology Co. Ltd. Beijing, China). These primers were experimentally validated using a panel of templates including in the common wheat Chinese Spring (CS), Norin 26 (NL26), *P. huashanica*, and the wheat-*L. mollis* 4Ns (4D) disomic substitution line M862.

## 3 Results

### 3.1 In suit hybridization analyses of M892 and M956

Two wheat-*L. mollis* translocation lines (M892 and M956) were derived from F_4_ generation of a hybridization between Norin26 + 3C (gametocidal chromosome 3C of *Aegilops triuncialis*) addition line and the wheat-*L. mollis* 4Ns addition line M852 (2*n* = 42 T.a + 2 L.m). The parental line M852 was generated through the cross between the common wheat cultivar 7182 and the wheat-*L. mollis* partial amphiploid M47 (2*n* = 56 = 42 T.a + 14 L.m).

**Figure 1:**
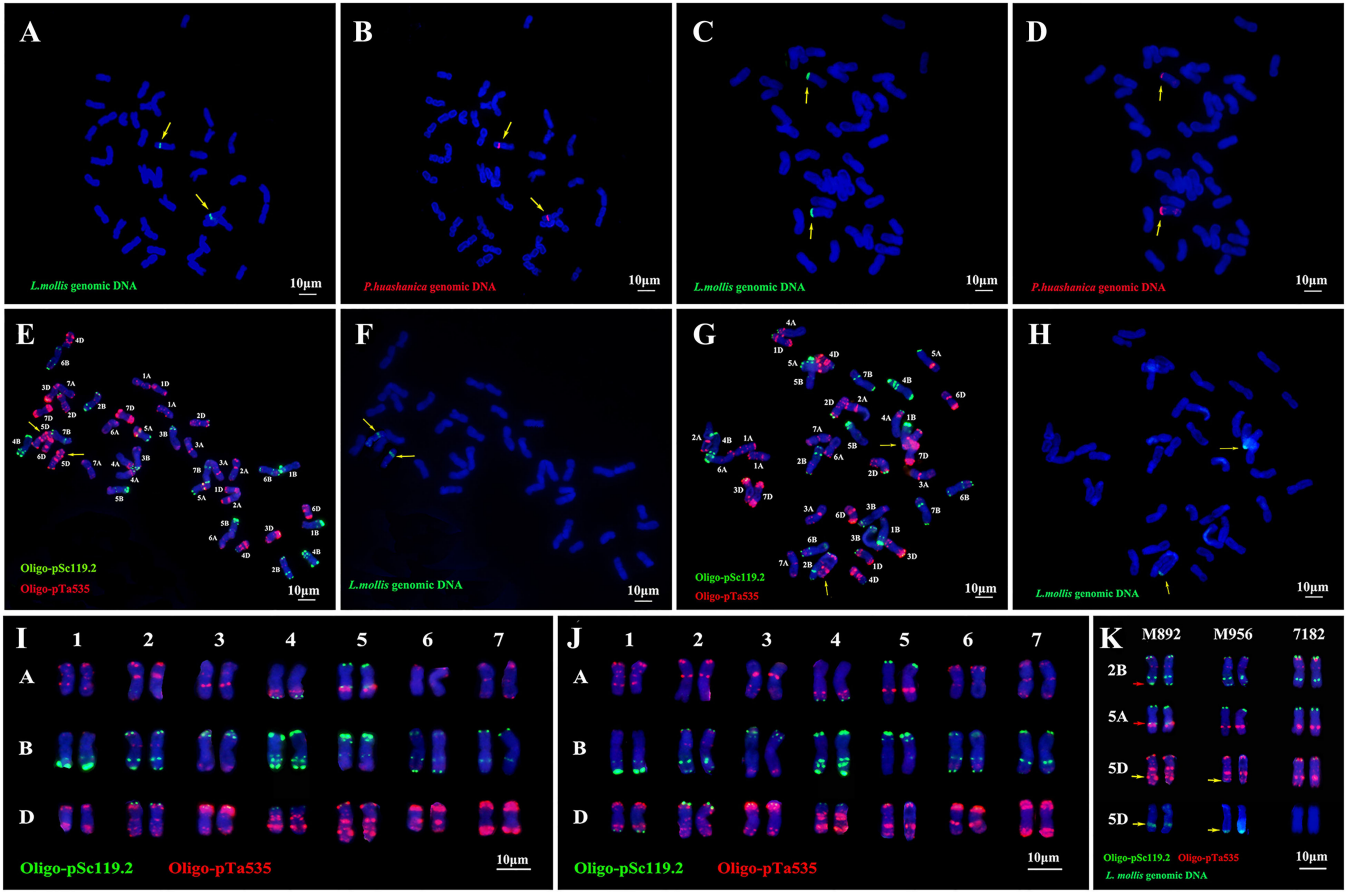
M892 and M956 identified by GISH with genomic DNA as probes and FISH with Oligo-pSc119.2 (green) and Oligo-pTa535 (red) as probes. **(A)** GISH analysis with *L. mollis* genomic DNA (green) as a probe in M895. **(B)** GISH analysis with *P. huashanica* genomic DNA (Red) as a probe in M895. **(C)** GISH analysis with *L. mollis* genomic DNA (green) as a probe in M956. **(D)** GISH analysis with *P. huashanica* genomic DNA (Red) as a probe in M956. **(E)** FISH analysis in M892. **(F)** Sequential FISH-GISH analysis in M892. **(G)** FISH analysis in M956. **(H)** Sequential FISH-GISH analysis in M956. **(I)** FISH karyotype of M892. **(J)** FISH karyotype of M956. **(K)** FISH karyotype variation of 1D, 2B, 5A chromosomes and 5D chromosome sequential FISH-GISH analysis in M892, M956 and their parent 7182. The yellow arrows referred to alien chromosomes in **(A-H)**. The red and yellow arrows referred to chromosome variation and a foreign fragment of 5D chromosome. Chromosomes were counterstained with DAPI (blue). Scale bar = 10 μm.

GISH analysis of root tip somatic chromosomes using *L. mollis* (green) and *P. huashanica* (red) genomic DNA probes revealed that both M892 and M956 (2*n* = 42) exhibited distinct 4Ns chromosomal translocations. In M892, dual-color fluorescence signals were localized to a mid-arm interstitial fragment on a pair of homologous chromosomes (**Figures 1A, B**). In contrast, M956 displayed terminal hybridization signals (green and red) at the distal end of a pair of chromosomes (**Figures 1C, D**).

Distinct integration patterns of translocation architecture were revealed through sequential FISH-GISH analysis using Oligo-pSc119.2 (green), Oligo-pTa535 (red), and *L. mollis* genomic DNA probes. M892 harbored an *L. mollis* Ns chromosome fragment (about 10% of total chromosome length), which was inserted interstitially into the 5DL arm of wheat chromosome 5D, thus forming a T5DL-4Ns-5DL·5DS insertional translocation (**Figures 1E, F**). M956 exhibited a terminal translocation with the Ns fragment (about 8% of chromosome length) fused to the 5DL terminus, classified as a T4Ns-5DL·5DS terminal translocation line (**Figures 1G, H**).

### 3.2 Karyotype variation analysis of M892 and M956

To elucidate chromosomal structural variations in lines M892 and M956, comparative FISH analysis was performed using Oligo-pSc119.2 (green) and Oligo-Ta535 (red) probes, with common wheat parent 7182 as the reference genotype (**Figures 1I, K**). The 5DS chromosomal signals showed complete conservation between M892 and 7182. However, M892 exhibited two distinct red signals in the mid-arm region of 5DL, a feature absent in 7182 (**Figure 1K**). Sequential FISH-GISH analysis confirmed that these additional signals corresponded to exogenous translocation fragments. Morphometric analysis demonstrated a 10% elongation by inserting 4Ns fragment of the 5DL chromosome arm in M892 compared to 7182 (**Figure 1K**). Meanwhile, 5DS signals remained consistent between M956 and 7182, the signal of the remaining 5DL portion aligns more closely with that of 5DL in 7182, whereas the exogenous fragment at the 5DL end shows no signal. The distinct FISH signals of the 4Ns fragments in M892 and M956 imply potential structural or compositional differences in the introgressed *L. mollis* chromatin between the two lines.

Whole-genome FISH karyotyping revealed structural polymorphisms in other chromosomes. For instance, M892 exhibited a terminal red signal on 1DL, while M956 showed colocalized red-green signals at this locus. Moreover, both M892 and M956 displayed novel terminal green signals on 2BL. M892 contained an additional green signal nearing the mid-arm red signal on 5AL, whereas M956 maintained conserved 5A signal patterns (**Figure 1K**). The remaining chromosomes showed complete signal pattern conservation across all three genotypes (**Figures 1I, J**). Notably, the 4Ns fragment insertion in M892 induced secondary structural rearrangements in chromosomes 1D, 2B, and 5A, while M956 exhibited more localized variation affecting chromosomes 1D and 2B.

### 3.3 GenoBaits^®^WheatplusPh array analysis for M892 and M956

**Figure 2:**
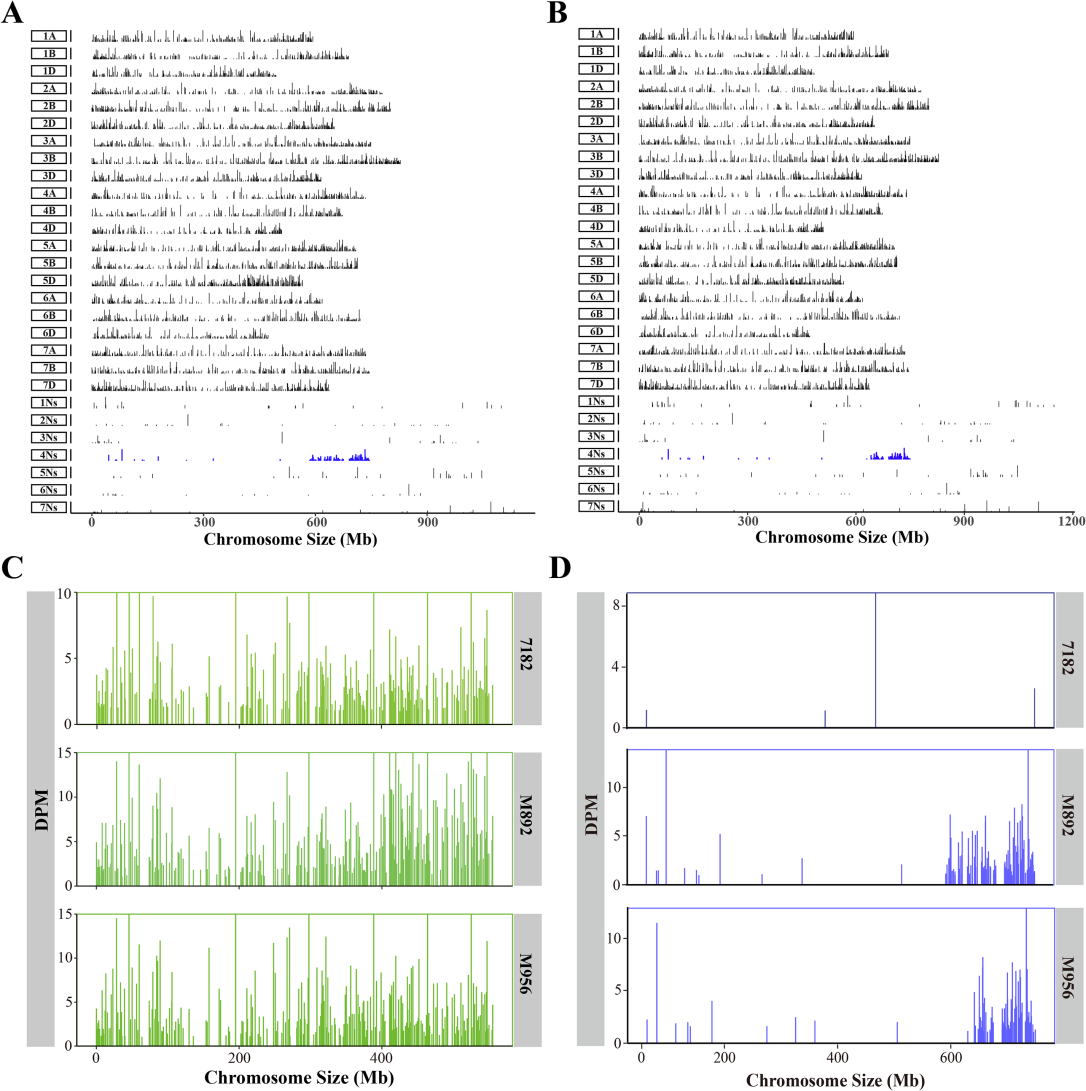
Analysis of GenoBaits^®^WheatplusPh array. **(A)** Whole genome analysis of M892. **(B)** Whole genome analysis of M956. **(C)** The chromosome 5D comparison with of M892 and M956 and their parent cv. 7182. **(D)** The chromosome 4Ns comparison with M892, M956 and their parent cv. 7182.

To determine the chromosomal compositions of M892 and M956, genome-wide analysis was performed using the GenoBaits^®^WheatplusPh array (**Supplementary Table 2**; **Figure 2**). In M892, uniform hybridization signals were detected across all 21 wheat chromosomes. Notably, chromosome 4Ns exhibited a distinct signal enrichment on its long arm (4NsL), while the remaining Ns chromosomes showed only sporadic signal distribution (**Figure 2A**). Meanwhile, M956 displayed evenly distributed signals across all wheat chromosomes, and pronounced signal accumulation was specifically localized to the 4NsL region (**Figure 2B**). These observations indicate that both lines carry exogenous fragments derived from the 4NsL segment of *L. mollis*.

Comparative analysis of chromosome 5D showed that conserved signal patterns were revealed between M892, M956 and their parent 7182. Notably, no deletions were detected in critical regions. M892 retained intact chromatin at the mid-position of 5DL, and M956 preserved the terminal region of 5DL (**Figure 2C**). Structural characterization further classified M892 as a wheat-*L. mollis* T5DL-4NsL-5DL·5DS insertional translocation line, and M956 as a wheat-*L. mollis* T4NsL-5DL-5DS terminal transition line, highlighted their distinct genomic architectures.

### 3.4 Responses to stripe rust and FHB of M892 and M956

**Figure 3:**
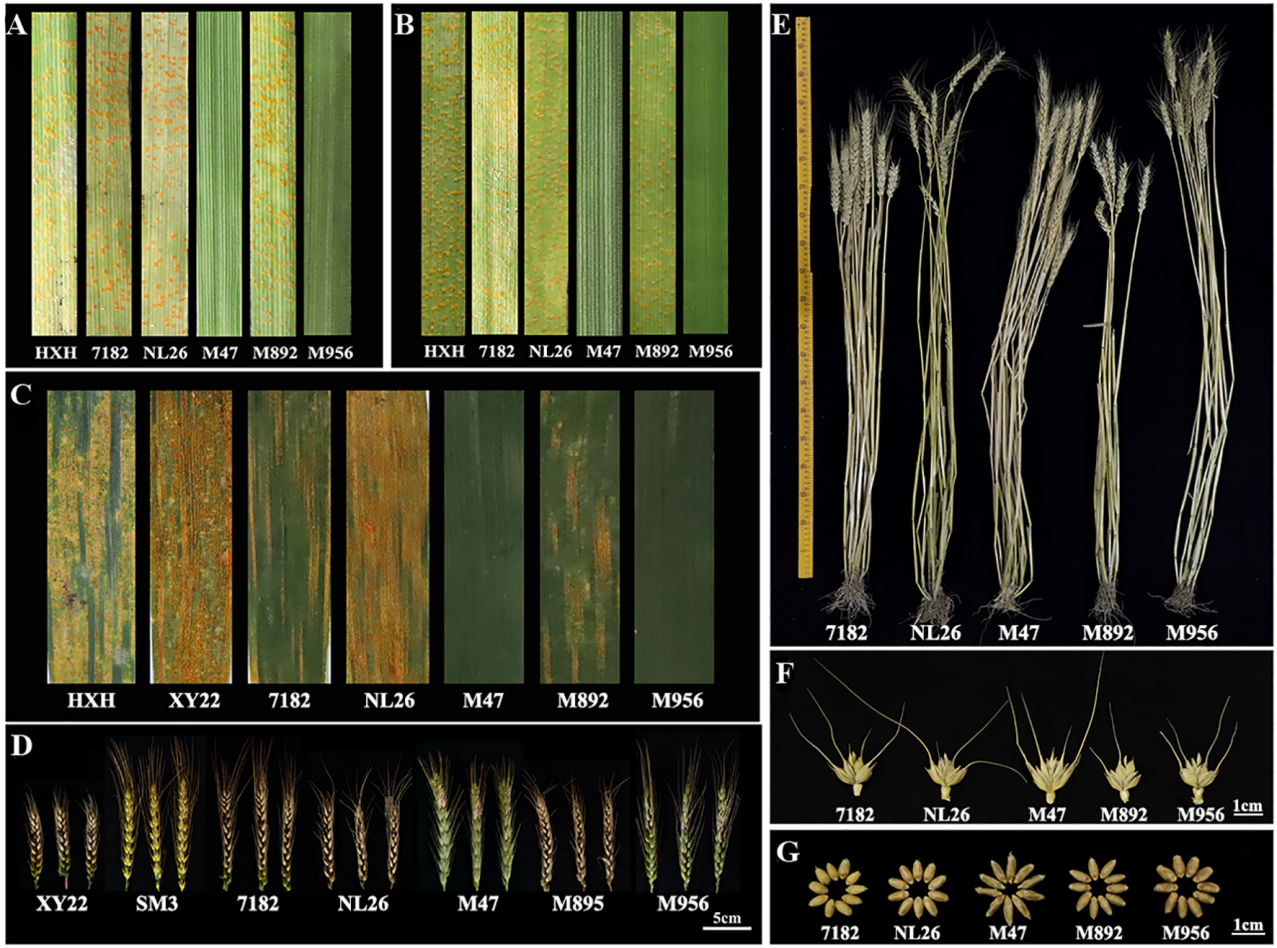
Evaluation of disease resistance and agronomic traits in M892, M956 and their parents. **(A, B)** Stripe rust identification to CYR32 and CYR34 *Pst* races at seedling stage. **(C)** Stripe rust identification to the mix of CYR32 and CYR34 *Pst* races at adult stage. **(D)** FHB identification to Ph1 *F. graminearum* strain. **(E)** Adult plant. **(F)** Spikelets. **(G)** Seeds.

The wheat-*L. mollis* translocation lines M892 and M956, as well as their parents were evaluated for resistance to *Pst* races CYR32 and CYR34 at seedling stage (**Figures 3A, B**; **Supplementary Table 3**). The susceptible control HXH consistently displayed maximum susceptibility (IT = 4). The two recurrent parents 7182 and NL26 exhibited high susceptibility to races, while the donor parent M47 demonstrated near-immunity (IT = 0). Notably, M892 showed differential responses with high susceptibility to CYR32 (IT = 4) and moderate susceptibility to CYR34 (IT = 3). In contrast, M956 exhibited complete immunity to both races (IT = 0), with no visible uredinia or necrotic lesions.

Adult-plants resistance were assessed under natural infection with mixed races of *Pst* (CYR32/CYR33/CYR34) over two consecutive growing seasons, and revealed distinct resistance patterns (**Figure 3C**; **Supplementary Table 3**). The controls HXH and XY22 showed high susceptibility, while 7182 and NL26 displayed moderate susceptibility (IT = 3) and high susceptibility (IT = 4), respectively. The donor parents *L. mollis* and M47 maintained complete immunity (IT = 0) with restricted uredial development and hypersensitive cell death. M892 demonstrated moderate susceptibility (IT = 2^+^-3), while M956 exhibited high-level resistance (IT = 0-1) comparable to *L. mollis* and M47. These consistent results at developmental stages confirmed the successful introgression of stripe rust resistance genes from *L. mollis* into M956.

Field evaluations of *F. graminearum* PH1 resistance during the 2020-2022 growing seasons revealed significant genotypic variation in 21 days post-inoculation (**Figure 3D**; **Supplementary Table 4**). Susceptible control XY22 showed high infected spikelets (ISR) with a severity level (SL) ≥3.4, while resistant control SM3 maintained SL = 1. The recurrent parents 7182 and NL26 were susceptible (ISR = 76.09 ± 0.1%, SL = 4) and moderately susceptible (ISR = 51.98 ± 6.59%, SL = 3.3), respectively. Notably, M892 remained as susceptible (ISR = 77.29 ± 0.2%, SL = 4) comparable to 7182, whereas M956 showed significantly enhanced resistance compared to the wheat parents but had less resistance (ISR = 13.46 ± 2.96%, SL = 1.6 ± 0.2) than parent M47 (ISR = 4.49 ± 0.28%, SL = 1.0). This strong resistance phenotype, coupled with pedigree analysis, confirms the successful transfer of *L. mollis*-derived FHB resistance genes into M956.

### 3.5 Agronomic traits evaluation of M892 and M956

Ten agronomic traits were measured for M892, M956 and their parents (**Figures 3E–G**; **Supplementary Table 5**). The results show that M892 had a slightly lower plant height than parent 7182. However, for the other parents, there were significant differences. Similarly, M956 had a significantly lower plant height compared to other parents. In terms of panicle length and spikelet number, M892 and M956 were similar to their parents. However, the number of spikelets in M892 and M956 was relatively few and the length of these spikelets was close to that of the three parents. Among the tested varieties, M956 had the highest number of grains per panicle, followed by M892. Moreover, M892 had the heaviest thousand-grain weight, with both M956 and M892 outperformed their parent varieties in this regard. The grains of M956 were longer (oblong), whereas those of M892 were rounded (oval), and both varieties were better in terms of grain plumpness. Overall, M892 and M956 possess favorable grain characteristics, and M892 also has desirable short stems.

### 3.6 Analysis of 4NsL fragment size and preliminary exploration of disease resistance gene regions

Comparative analysis of 4Ns chromosomal loci through sequencing depth (DPM) revealed that distinct 4NsL fragment sizes existed between the translocation lines (**Figure 2D**). The M892 line contained a 162 Mb fragment spanning the 584-746 Mb region, and M956 possessed a 98 Mb fragment (648-746 Mb), which was 60.5% of the length of the M892 fragment. Importantly, resistance screening for stripe rust and FHB identified co-localized resistance loci within the 648-746 Mb region of the 4Ns chromosome. This overlapping interval suggests that there may be the potential presence of linked or pleiotropic disease resistance genes in the conserved 98 Mb segment shared by the two translocation lines.

### 3.7 Transcriptome data analysis and differential expression patterns in 5D chromosome

RNA-seq of 45 biological samples generated 1.47 billion raw reads (440.82 Gb), with an average of 32.76 million high-quality reads per sample (Q20 > 96%, Q30 > 90%) (**Supplementary Table 6**). After quality filtering, clean reads were aligned to a combined reference genome comprising *Triticum aestivum* (IWGSC RefSeq v2.1) and *Puccinia striiformis* (Pst134E36_v1_pri), yielding 1,544,884,33 mapped reads (89.61%) (**Supplementary Table 6**). Subsequently, 51,260 high-confidence wheat genes were identified by stringent screening (FPKM ≥ 1 in at least one sample) to remove *P. striiformis*-homologous genes. These genes were retained for differential expression analysis to systematically investigate transcriptional perturbations caused by exogenous chromosomal segment introgression in the wheat background.

**Figure 4:**
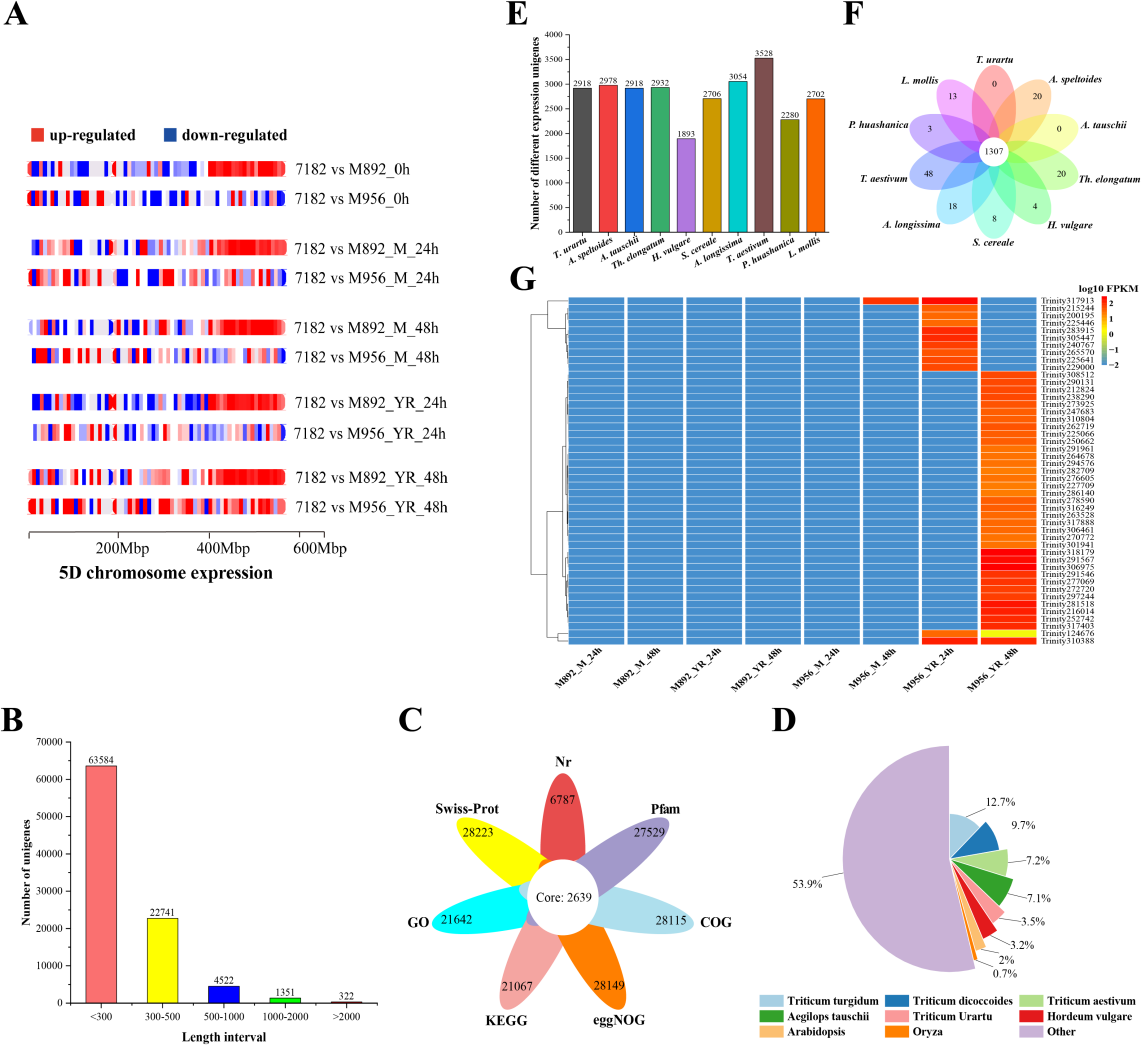
Transcriptome sequencing analysis. **(A)** The 5D chromosome distribution of DEGs among M892, M956 and 7182. **(B)** Lm#4NsL unigenes quantity distribution of different lengths. **(C)** Venn-diagram of different databases annotation for Lm#4NsL unigenes. **(D)** Pie-diagram of Lm#4NsL unigene in Nr databases compared to different species. **(E)** Histogram of the distribution for Lm#4NsL unigene in the genomes of 10 wheat and its relatives. **(F)** Venn-diagram of Lm#4NsL candidate genes have orthologs located on chromosomes of homoeologous group 4 among related species. **(G)** Heatmap diagram of Lm#4NsL disease resistance candidate unigenes expression.

To explore the influence of the translocated 4NsL fragments on 5D chromosome in lines M892 and M956, we compared the differentially expressed genes (DEGs) between two translocation lines and the wheat parent 7182 under both mock (water-treated) and *P. striiformis*-inoculated conditions at three time points (0 h, 24 h, 48 h). The analysis revealed distinct spatial-temporal expression patterns between the two lines (**Figure 4A**). In M892, most DEGs on the 5DS arm were down-regulated throughout all time points and treatments. Conversely, a mid-to-terminal 5DL gene cluster exhibited sustained up-regulation, which indicates localized transcriptional activation. M956 displayed a contrasting regulatory pattern. Specifically, 5DL-arm DEGs were predominantly up-regulated only at 48 h post-inoculation. Under other conditions, the number of down-regulated genes was consistently similar to or greater than that of up-regulated counterparts. These observations suggest that the insertion of the 4NsL fragment in M892 has a significantly greater impact on the transcriptional activity of chromosome 5D than the terminal translocation in M956.

### 3.8 Reference free transcriptome analysis and functional annotation

After sequence alignment of the mixed-genome dataset, a total of 3,433,076 reads (10.39%) remained unmapped. The *de novo* assembly of unmapped reads from the M892 and M956 libraries produced 395,502 transcripts, which were further clustered into 314,126 non-redundant unigenes. Following the removal of wheat-derived sequences, we identified 92,520 *L. mollis*-specific unigenes (29.6 Mb assembly) with contig N50 of 289 bp, maximum length 9,571 bp, and GC content 46.59% (**Supplementary Table 7**). About 69% of unigenes are less than 300 bp, with 22741 unigenes of 300-500 bp, 4,522 unigenes of 500-1,000 bp, and 1,673 unigenes of more than 1,000 bp (**Figure 4B**).

For functional elucidation of these uncharacterized alien unigenes, comprehensive annotation was performed against seven databases (**Figure 4C**): Nr, Swiss-Prot, GO, KEGG, eggNOG, COG, and Pfam. Among the 92,520 unigenes, 7.34% (6,787) matched sequences in the Nr database, while Swiss-Prot, GO, KEGG, eggNOG, COG, and Pfam annotations accounted for 30.50% (28,223), 23.39% (21,642), 22.77% (21,067), 30.42% (28,149), 30.39% (28,115), and 29.75% (27,529), respectively. Moreover, 2,639 unigenes (2.85%) were commonly annotated across all databases, which highlighted conserved functional domains.

Comparative analysis against the Nr database revealed phylogenetic relationships of the alien unigenes, with the highest homology to *Triticum turgidum* (12.7%), followed by *Triticum dicoccodes* (9.7%), *Triticum aestivum* (7.2%), *Aegilops tauschii* (7.1%), and other species including *Triticum urartu* (3.5%), *Hordeum vulgare* (3.2%), *Arabidopsis* (2.0%), and *Oryza sativa* (0.7%) (**Figure 4D**; **Supplementary Table 8**). These annotations provide critical insights into the putative roles of *L. mollis* 4NsL-derived unigenes, particularly in identifying candidate genes associated with disease resistance and stress adaptation.

### 3.9 Differential expression unigenes analysis of Lm#4NsL

Comparative transcriptomic analysis was performed on M892 and M956 to identify Lm#4NsL-specific unigenes, and DEGs were assessed at multiple time points (**Supplementary Table 9**). At the same time points, M892 had significantly more DEGs than M956. In M892, up-regulated unigenes consistently outnumbered down-regulated ones across all time points. Conversely, in M956, down-regulated unigenes marginally predominated at 24 h, but this pattern reversed at 48 h, with a substantial dominance of up-regulated unigenes (**Supplementary Figure 1A**). Obviously, both lines had significantly more up-regulated than down-regulated unigenes at 48 h.

Intersection analysis showed that there were 184 shared DEGs between M892 and M956 at 24 h, including 72 up-regulated, 50 down-regulated, and 62 with opposing expression trends. This number decreased to 112 shared DEGs at 48 h, where 83 were up-regulated, 14 were down-regulated, and 15 inversely regulated genes (**Supplementary Figures 1B–D**). Thus, the comprehensive DEG profiling establishes that Lm#4NsL in M892 exhibits enhanced transcriptional responsiveness compared to M956, particularly in sustaining activation signals during the progression of infection.

### 3.10 Development and validation of Lm#4NsL chromosome-specific molecular markers

Based on the previously identified Lm#4NsL differential unigenes, a comparative analysis between M956 and M892 was conducted, which identified 11,368 unigenes that were exclusively differentially expressed in M956. Cross-species genomic comparisons with ten wheat-related species revealed varying degrees of homology, with the highest homology (3,528 unigenes) observed in wheat (*Triticum aestivum*) and the lowest (1,893) in barley (*Hordeum vulgare*) (**Figure 4E**). Subsequent comparative screening of Lm#4NsL-specific unigenes in M956 across species identified 3 unique to *P. huashanica*, 13 unique to *L. mollis*, and 19 shared between the two species (**Figure 4F**). These conserved sequences served as the basis for developing Lm#4NsL-specific molecular markers in M956.

**Figure 5:**
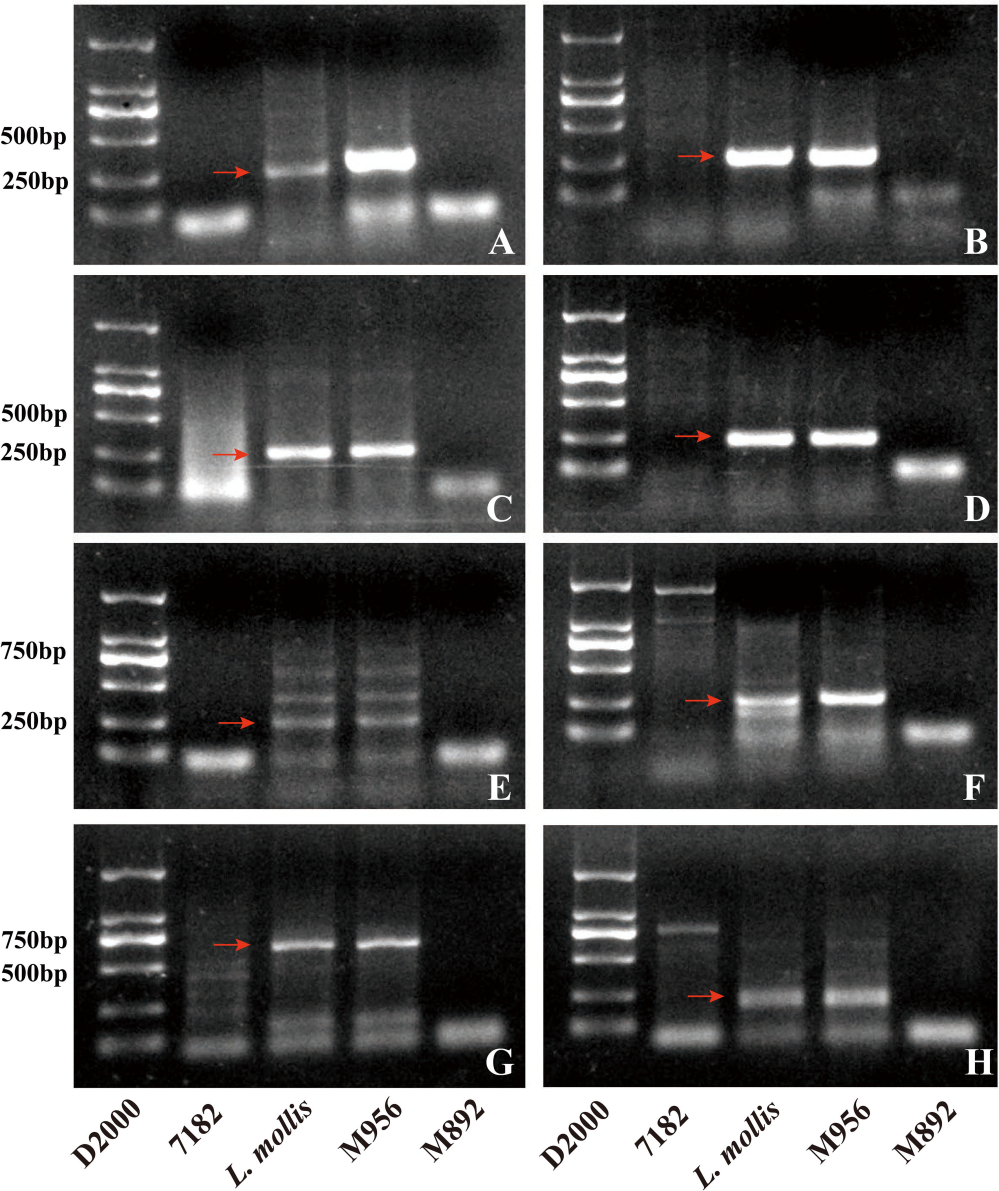
Candidate special primers for Lm#4NsL of M956 were designed by RNA-seq DEGs. **(A)***Lm4-124676*. **(B)***Lm4-198207*. **(C)***Lm4-206959*. **(D)***Lm4-237228*. **(E)***Lm4-250662*. **(F)***Lm4-254212*. **(G)***Lm4-271348*. **(H)***Lm4-284634.* The red arrow represents the specific band of the Lm#4NsL.

Across seven databases, functional annotation identified 80 disease resistance-associated unigenes within the 4NsL fragment of both M892 and M956. Expression profiling narrowed this set to 47 candidate disease-resistant unigenes in M956 Lm#4NsL, characterized by high expression in M956 and low expression in M892 (**Figure 4G**; **Supplementary Table 10**). From these, combining 19 unigenes were mapped to *L. mollis* and *P. huashanica* genomes (**Supplementary Table 11**). Then, 11 candidate unigenes with 20% similarity to the wheat genome were selected for marker development (**Supplementary Table 12**). This process yielded eight Lm#4NsL-specific markers (*Lm4-124676*, *Lm4-198207*, *Lm4-206959*, *Lm4-237228*, *Lm4-250662*, *Lm4-254212*, *Lm4-271348*, *Lm4-284634*) were identified in M956 (**Figure 5**). Among them, compared to *L. mollis* and M956, three markers (*Lm4-254212*, *Lm4-271348* and *Lm4-284634*) produced differential amplicon sizes in parent line 7182, while other five markers exhibited exclusive amplification in *L. mollis* and M956. Notably, *Lm4-124676* and *Lm4-250662* were associated with disease resistance (**Figure 4G**; **Supplementary Table 12**).

**Figure 6:**
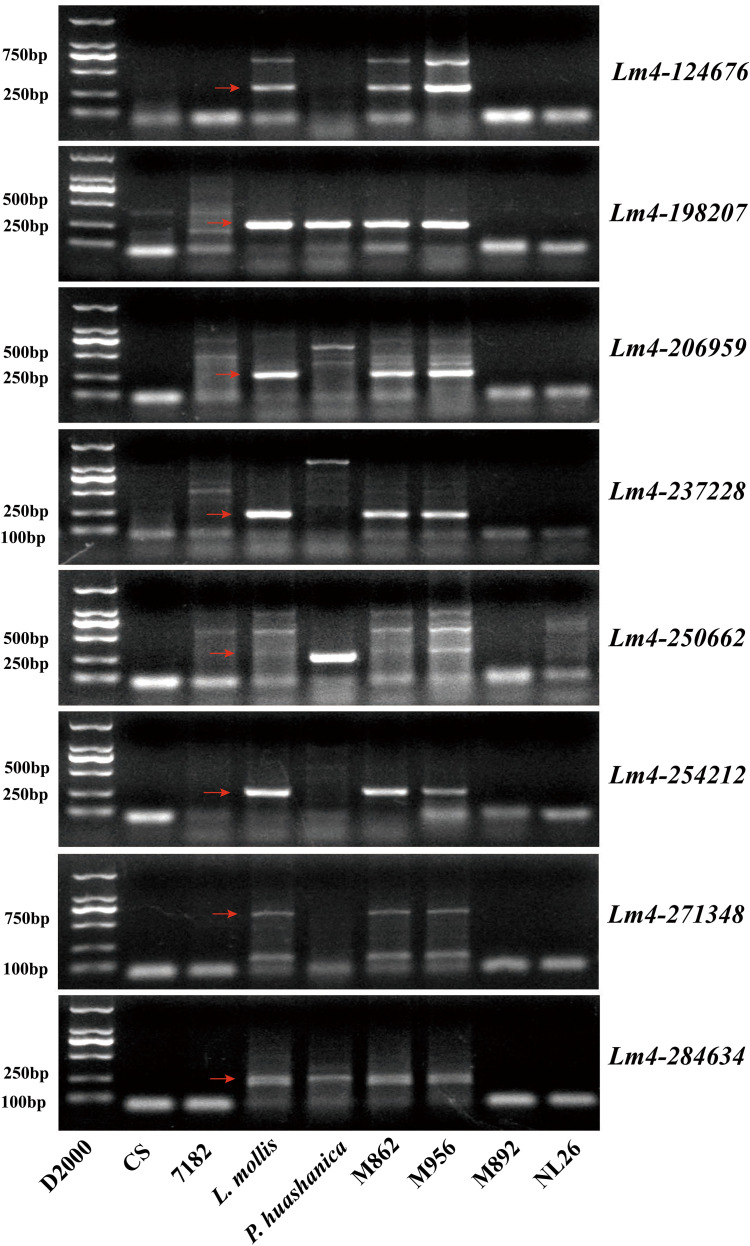
Validation of the Lm#4NsL fragment-specific markers in M956. The red arrow represents the specific band of the Lm#4NsL.

Specificity validation using common wheat China Spring (CS), Nonglin26 (NL26), *P. huashanica*, and the wheat-*L. mollis* 4Ns (4D) disomic substitution line M862 confirmed marker fidelity (**Figure 6**; **Table 1**). All eight markers failed to amplify in NL26 but showed identical banding patterns in M862, *L. mollis*, and M956. Four markers (*Lm4-198207*, *Lm4-206959*, *Lm4-237228*, *Lm4-250662*) generated size-polymorphic bands in CS or parent 7182 compared to M862. Crucially, four markers (*Lm4-124676*, *Lm4-254212*, *Lm4-271348*, *Lm4-284634*) demonstrated absolute specificity, amplifying only in *P. huashanica*, *L. mollis* and its derivatives. Overall, we obtained a total of five partially specific and three fully specific markers of *L. mollis*, which providing a robust toolkit for tracking the *L. mollis* 4NsL chromosomal segment and identifying disease-resistant germplasm in wheat breeding programs.

**Table 1:** Informatica of special molecular markers for Lm#4NsL segment in M956.

Marker	Primer (5’-3’)	Tm (°C)
*Lm4-124676*	F:CTGTTGCATCTCCTGTCGGT	60
	R: AGTATGGACAAGGCTCTGCG	
*Lm4-198207*	F:GCCCGCTATAACCCAGATCC	60
	R:CCACGGTTGTCCATGTAGCT	
*Lm4-206959*	F:CGCTCTTGACTCACTCTCACA	61
	R:CCTACAGTGCTCCTCCCCG	
*Lm4-237228*	F:ACAAGGCATCGACATCAATCAAAG	62
	R:GCTCGACGCGGACTTTTGAG	
*Lm4-250662*	F:CATGGCAGCAGGATTTAAAATGTTC	60
	R:GACGCAGAAACGGGCAAAG	
*Lm4-254212*	F:GGAAGTACAAAACTCCCTCGGA	60
	R:AACGTTGCCGACCAAAGAAG	
*Lm4-271348*	F:CCACTTTGAACCGACCCAGT	60
	R:GGACTAAAATGAGGGCCGGA	
*Lm4-284634*	F:AGCAGAAGGATCTCAACGCC	62
	R:ACTCATGCTGCCGCCCTC	

## 4 Discussion

Chromosome engineering techniques provide an effective approach for creating heterologous translocation lines in wheat, which are crucial for expanding genetic variation and improving agronomic traits in common wheat. These translocation lines have been extensively adopted in wheat breeding due to their desirable traits and minimal genetic linkage resistance. A landmark achievement in this field is the wheat-rye 1RS·1BL translocation line, which introduced critical disease resistance genes (*Yr9*, *Lr26*, *Sr31*) (Rayburn and Carver, 1988; Singh et al., 1990) and has been incorporated into about 30% of post-2000 wheat cultivars (Wang et al., 2017). Similarly, the wheat-*Dasypyrum villosum* 6VS·6AL translocation, harboring the broad-spectrum powdery mildew resistance gene *Pm21*, has become a cornerstone of wheat breeding in Chinese, contributing to over 40 commercial varieties (Qi et al., 1996; Xing et al., 2018; Zhang et al., 2023). Additional translocations, including wheat-*Aegilops ventricosa* T2N^v^S·2AS (Gao et al., 2021), wheat-*Agropyron cristatum* 6PS (Zhang et al., 2021), wheat-rye 6RL (Li et al., 2020; Ren et al., 2022), and wheat-*Thinopyrum ponticum* T7DS·7DL-7el_2_L (Li et al., 2023), which showed resistance to various pathogens such as powdery mildew, stripe rust, FHB, and stem rot, respectively.

Despite progress, reports on wheat-*L. mollis* translocation lines are still limited. The 6BS·6NsS additional translocation line M13063A-1 and T4BS·1NsL lines M11006A2/A3 exhibit adult-stage stripe rust resistance (Du et al., 2020). Although earlier lines WL24-4 (5DS·5DL-TTr) and WL21-46 (5DS·5DL-ITr) showed different stripe rust responses (Li et al., 2015), their translocation structures remained ambiguous. In this study, we integrated sequential FISH-GISH and the GenoBaits^®^WheatplusPh array analyses to clarify that M956 is a T4NsL-5DL·5DS terminal translocation and M892 of a T5DL-4NsL-5DL·5DS insertion translocation (**Figures 2**, **3**). Since WL24-4, WL21-46, M956, and M892 were generated from the same hybrid combination, based on sequential FISH-GISH results, it is speculated that WL24-4 is a T4Ns-5DL·5DS terminal translocation line, and WL21-46 is a wheat-*L. mollis* T5DL-4NsL-5DL·5DS insertion translocation line. Notably, M956 and the 4Ns(4D) substitution line M862 displayed broad-spectrum stripe rust resistance at both the seedling (**Figures 3A, B**) and adult stages (**Figure 3C**), as well as good resistance to FHB (**Figure 3D**) (Du et al., 2022). This implies that the 4NsL fragment likely contains genes responsible for stripe rust and FHB resistance. The screening and identification of M956 further refined the segments of the 4Ns chromosome that are associated with these disease resistances, thus laying a solid foundation for exploration and utilization of disease resistance genes in the future.

Beyond pathogen resistance, translocation lines significantly improved yield-related traits. The wheat-*Agropyron cristatum* T1AS-6PL-1AS·1AL insertion translocation line Pubing 2978 and the T5BL·5BS-6PL terminal translocation line WAT650a increased the number of grains per panicle. Molecular marker analysis indicated that these two translocation lines share overlapping 6PL fragments and carry the multigrain gene located at 6PL (0.35-0.42) (Zhang et al., 2016, 2019). Moreover, in the wheat-*Dasypyrum villosum* T6VS·6DL translocation line PM97033, the abnormal transcription of *DvGW2*, a homologous gene of the rice grain weight gene *GW2* on the 6VS chromosome arm, contributed to an increase in grain width and thousand-grain weight (Feng et al., 2021). M892, M956 and their parent wheat-*L. mollis* disomic substitution line M862, also show promise in this regard, both translocation lines having heavier grain weights, and GenoBaits^®^WheatplusPh array identification reveals that some fragments of 4NsL was overlapped (**Figure 2D**). This suggests that 4NsL may contain genes associated with increasing grain weight, making it a valuable resource for gene mining and wheat breeding.

The biological characteristics of wheat distant hybrids remains poorly understood. Previous studies indicate that the introduction of whole chromosomes or large chromatin segments from wheat-related species, along with their inherent deleterious haplotypes, often leads to yield impairment or undesirable traits (Kerber and Dyck, 1973; Chen et al., 2005; Hurni et al., 2014; Rey et al., 2018). This phenomenon may arise not only from the introduction of exogenous chromatin but also from the superposition of unexpected genetic, epigenetic and gene regulatory effects that may induce reorganization of the transcriptome, proteome, metabolome, and phenome (Lynch and Conery, 2000; Prince and Pickett, 2002; Duarte et al., 2006; Buggs et al., 2011). Emerging evidence highlights that the interactions between donor and recipient genomes can affect plant phenotypes (Chaudhary et al., 2009), such as the suppression of pathogen resistance genes by host genomic elements (Kerber and Aung, 1999; Chen et al., 2005). Consequently, incompatible interactions between donor- and acceptor-derived gene products may negatively impact plant performance (Hurni et al., 2014). In this study, both M862 and M956 carry 4NsL chromosomal segments. Especially, the 98 Mb 4NsL fragment in M956 is entirely encompassed within the 4NsL region of M892. Although M892 is theoretically expected to have equal or greater disease resistance, M892 exhibited high susceptibility to stripe rust and FHB, whereas M956 demonstrated strong resistance. This paradox enabled initial localization of resistance genes to the 648–746 Mb region of chromosome 4Ns. Three hypotheses are proposed to explain this divergency: (I) In M892, the interstitial insertion of 4NsL into 5DL may induce significant up-regulation of 5D chromosomal genes, which may repress the expression of resistance gene derived from 4NsL. Conversely, the terminal fusion of 4NsL to 5DL in M956 minimizes interference from 5D chromosomal elements, thus allowing normal expression of resistance genes. (II) During selfing, introgressed segments may undergo structural rearrangements. In M892, frameshift mutations or premature termination codons introduced into resistance loci during recombination could disrupt gene function. By contrast, M956 may retain the intact resistance genes through precise recombination events. (III) Comparison of the 4NsL differential unigenes in M892 and M956 reveals that only 397 unigenes exhibited concordant expression patterns between the two lines, accounting for merely 3.8% of the total 10,443 differentially expressed unigenes identified in M956. Molecular markers for M956-specific unigenes further validated sequence-level divergence, suggesting that structural or regulatory variations in 4NsL may be responsible for the phenotypic differences.

The markedly divergent resistance phenotypes observed between M892 and M956, despite the shared introgressed segment, necessitate a deeper examination of the mechanisms that can govern the expression of alien genes in a wheat background. The integrated evidence from cytogenetic, genomic, and transcriptomic analyses points to a confluence of factors that likely underpin this discrepancy. The stark contrast in disease resistance is likely attributable to the profound influence of genomic context and structural integrity on alien gene expression, a phenomenon well-documented in wheat-alien introgression lines. Firstly, positional effects could have led to the silencing of resistance genes in M892’s interstitial translocation, where the embedded 4NsL fragment is flanked by wheat chromatin and may be subject to epigenetic repression or suppression by adjacent host genes—a mechanism evidenced by the variable expression of the *Thinopyrum ponticum*-derived *Lr19* gene in different genomic contexts (Prins et al., 2001). Secondly, structural rearrangements during the gametocidal chromosome-mediated recombination might have disrupted critical resistance loci in M892. Similar cases of resistance loss due to small, cryptic deletions at breakpoints have been reported in wheat-*Aegilops* sp*eltoides* translocations (Friebe et al., 1996). In contrast, the terminal translocation in M956 potentially preserved an intact and functional resistance complex. Finally, sequence-level divergence is strongly suggested by our transcriptome data, which revealed minimal overlap in 4NsL-derived unigene expression profiles and enabled the development of M956-specific markers. This indicates that the two lines carry structurally or regulatorily distinct versions of the introgressed chromatin, akin to the heterogeneity observed in wheat-rye translocation lines that leads to varied resistance phenotypes (Ren et al., 2022). Collectively, these mechanisms underscore that successful trait introgression relies not only on the physical transfer of chromatin but, critically, on its genomic position, structural integrity, and precise nucleotide sequence.

The development of chromosome-specific markers for wheat-related species lacking a reference genome has emerged as a key strategy in modern cereal genomics. Recent advances in transcriptome sequencing coupled with *de novo* assembly techniques have made it possible to systematically identify alien chromosomal segments in wheat hybrids (Li et al., 2017; Wang et al., 2018), although technical challenges remain in terms of marker specificity and genome complexity resolution. Notably, Wang et al. (2018) demonstrated the effectiveness of this approach by establishing 134 *Elytrigia intermedia* chromosome-specific markers by RNA-seq analysis, including three validated markers for the wheat-*Aegilops longissima* 1BL·1S^l^S translocation line. In this investigation, we extended this methodology to analyze the *Leymus mollis*-derived germplasm lines M892 and M956. Comparative transcriptomic profiling revealed eight robust markers specific to the Lm#4NsL chromosomal fragment in M956 (**Figure 6**). The disease resistance-associated markers *Lm4-124676* and *Lm4-250662* share sequence homology with domains of Ser/Thr protein kinase family and ribokinase (RBKS), respectively, implying that they are involved in pathogen-responsive signaling cascades (**Figure 4G**; **Supplementary Table 12**). These findings not only contribute to marker-assisted selection for wheat improvement but also provide a foundation for map-based cloning of novel *Yr* and *FHB* homologs within the 4NsL region.

## 5 Conclusions

In conclusion, a wheat-*L. mollis* T4NsL-5DL·5DS terminal translocation line M956 showed resistance to both stripe rust and FHB, with heavy grains and minimal effect on gene expression in wheat. M892, a wheat-*L. mollis* T5DL-4NsL-5DL·5DS insertion translocation line, has heavier grain weight and shorter stem. The screening and identification of these two translocation lines are of great research value in exploring and utilizing 4NsL gene of the resistance to stripe rust and head blight. In addition, they are valuable germplasm resources for enhancing disease resistance and improving yield in wheat.

## Data Availability

The data presented in the study are deposited in the National Center of Biotechnology Information (NCBI) repository, accession number PRJNA1266866.
