# Supplementary material for: A 4NsL chromosome segment from Leymus mollis confers stripe rust and Fusarium head blight resistance in wheat
Source: Front Plant Sci. 2025 Dec 1;16:1711424. doi: 10.3389/fpls.2025.1711424 (PMC12702952; doi:10.3389/fpls.2025.1711424)
Supplement: Supplementary file 2 [file DataSheet1.doc]

**
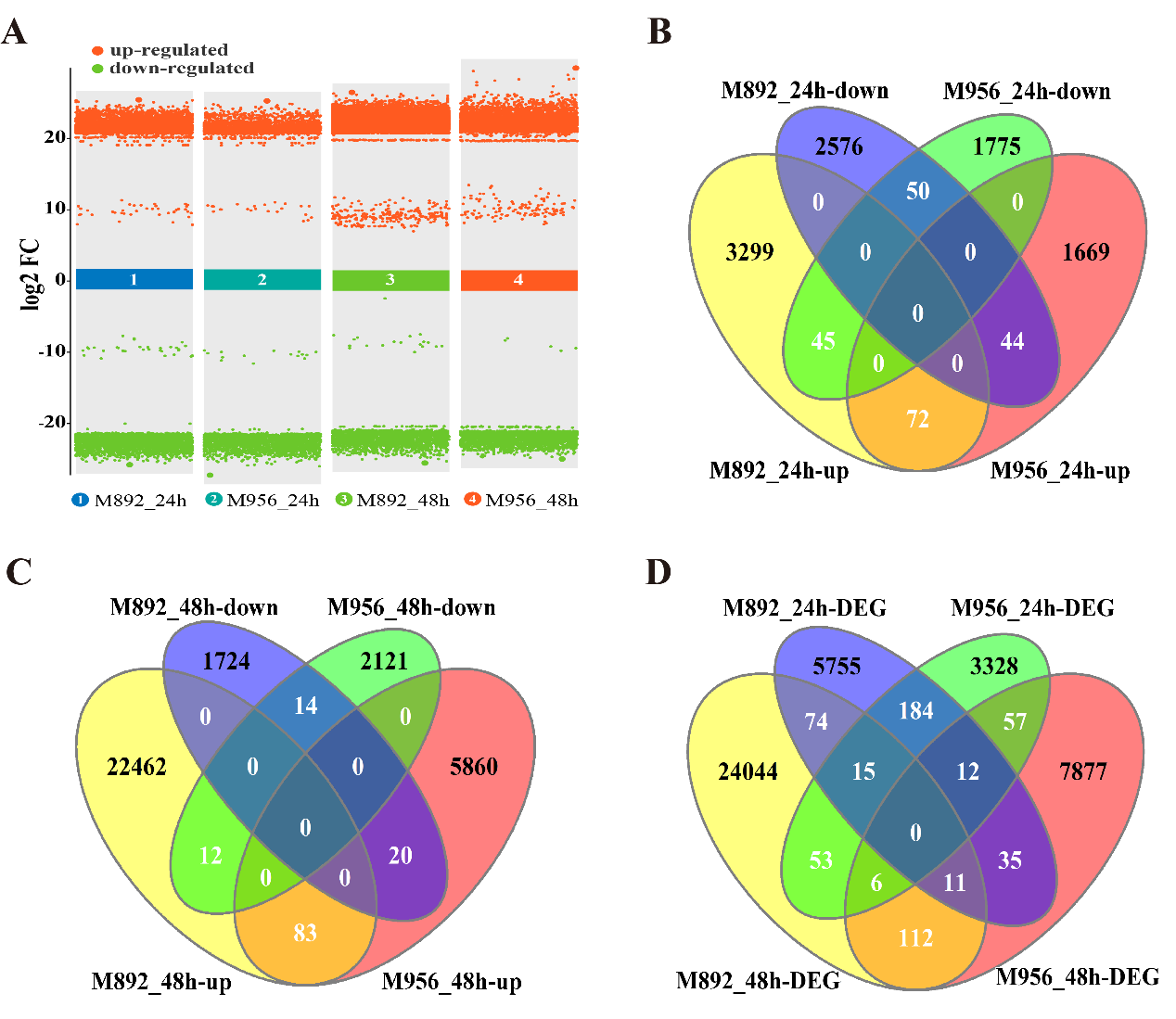
Supplementary Figure S1.** Differential unigenes expression of Lm#4NsL in M892 and M956. **(A)** Volcano plot of Lm#4NsL differential gene expression. **(B)** Venn plot of up- and down-regulated differential unigenes at 24 h for M892 and M956. **(C)** Venn plot of up- and down-regulated differential unigenes at 48 h for M892 and M956. **(D)** Venn plot of the differential unigenes between M892 and M956 at 24 h and 48 h.
